# Impact of community-initiated Kangaroo Mother Care on survival of low birth weight infants: study protocol for a randomized controlled trial

**DOI:** 10.1186/s13063-017-1991-7

**Published:** 2017-06-07

**Authors:** Sarmila Mazumder, Sunita Taneja, Suresh Kumar Dalpath, Rakesh Gupta, Brinda Dube, Bireshwar Sinha, Kiran Bhatia, Sachiyo Yoshida, Ole Frithjof Norheim, Rajiv Bahl, Halvor Sommerfelt, Nita Bhandari, Jose Martines

**Affiliations:** 1 Centre for Health Research and Development, Society for Applied Studies, New Delhi, India; 2 Child Health Division, National Rural Health Mission, Panchkula, Haryana India; 3 Department of Maternal, Newborn, Child and Adolescent Health, World Health Organization, Geneva, Switzerland; 4 Centre for Intervention Science in Maternal and Child Health, Department of Global Public Health and Primary Care, University of Bergen, N-5020 Bergen, Norway; 5 Norwegian Institute of Public Health, Oslo, Norway

**Keywords:** Community-initiated Kangaroo Mother Care, Low birth weight babies, Mortality

## Abstract

**Background:**

Around 70% neonatal deaths occur in low birth weight (LBW) babies. Globally, 15% of babies are born with LBW. Kangaroo Mother Care (KMC) appears to be an effective way to reduce mortality and morbidity among LBW babies. KMC comprises of early and continuous skin-to-skin contact between mother and baby as well as exclusive breastfeeding. Evidence derived from hospital-based studies shows that KMC results in a 40% relative reduction in mortality, a 58% relative reduction in the risk of nosocomial infections or sepsis, shorter hospital stay, and a lower risk of lower respiratory tract infections in babies with birth weight <2000 g. There has been considerable interest in KMC initiated outside health facilities for LBW babies born at home or discharged early. Currently, there is insufficient evidence to support initiation of KMC in the community (cKMC). Formative research in our study setting, where 24% of babies are born with LBW, demonstrated that KMC is feasible and acceptable when initiated at home for LBW babies. The aim of this trial is to determine the impact of cKMC on the survival of these babies.

**Methods/design:**

This randomized controlled trial is being undertaken in the Palwal and Faridabad districts in the State of Haryana, India. Neonates weighing 1500–2250 g identified within 3 days of birth and their mothers are being enrolled. Other inclusion criteria are that the family is likely to be available in the study area over the next 6 months, that KMC was not initiated in the delivery facility, and that the infant does not have an illness requiring hospitalization. Eligible neonates are randomized into intervention and control groups. The intervention is delivered through home visits during the first month of life by study workers with a background and education similar to that of workers in the government health system. An independent study team collects mortality and morbidity data as well as anthropometric measurements during periodic home visits. The primary outcomes of the study are postenrollment neonatal mortality and mortality between enrollment and 6 months of age. The secondary outcomes are breastfeeding practices; prevalence of illnesses and care-seeking practices for the same; hospitalizations; weight and length gain; and, in a subsample, neurodevelopment.

**Discussion:**

This efficacy trial will answer the question whether the benefits of KMC observed in hospital settings can also be observed when KMC is started in the community. The formative research used for intervention development suggests that the necessary high level of KMC adoption can be reached in the community, addressing a problem that seriously constrained conclusions in the only other trial in which researchers examined the benefits of cKMC.

**Trial registration:**

ClinicalTrials.gov identifier: NCT02653534. Registered on 26 December 2015 (retrospectively registered).

**Electronic supplementary material:**

The online version of this article (doi:10.1186/s13063-017-1991-7) contains supplementary material, which is available to authorized users.

## Background

Globally, around 15% of newborns are born with low birth weight (LBW) as a result of preterm birth or intrauterine growth retardation or both, and up to 70% of neonatal deaths occur in these infants [1]. In India, 28% of babies are born with LBW, and recent data derived from a study setting showed that 24% of babies are born with LBW [2, 3]. The causes of death in LBW neonates include respiratory and brain immaturity, hypothermia, hypoglycemia, and infection [1]. In addition, LBW infants are at high risk of impaired growth and development [4]. A recent review of available interventions suggested that breastfeeding, hygiene, antenatal corticosteroids to prevent preterm birth complications, case management of suspected infections, and hospital care of small babies that includes Kangaroo Mother Care (KMC) are the most effective interventions for improving survival of LBW infants [5].

KMC is an effective way to reduce mortality among LBW babies. It helps to meet babies’ needs for warmth, breast milk, protection from infection, safety, and love [6]. KMC as defined by the World Health Organization (WHO) is care of preterm and LBW infants where the mother keeps the baby in skin-to-skin contact (SSC) on her chest between her bare breasts continuously until the baby no longer wants to stay in that position, and exclusively breastfeeds the baby [6]. Authors of a recent Cochrane review reported a 40% relative reduction in mortality with this intervention compared with standard care in hospitalized infants with a birth weight <2000 g [7]. The review showed a 58% relative reduction in the occurrence of nosocomial infections or sepsis at discharge or at 40–41 weeks corrected gestational age, a shorter duration of hospital stay, and lower incidence of respiratory tract infections.

Prolonged SSC has been shown to provide effective thermal control for preterm and LBW babies. The review cited above showed a 76% relative reduction in the occurrence of hypothermia by hospital discharge or 40–41 weeks corrected gestational age [7]. KMC has been reported to increase the prevalence and duration of breastfeeding and exclusive breastfeeding, also in India [8–15]. A meta-analysis showed that infants receiving KMC gained more weight per day by the time of discharge from the birth facility and had a larger head circumference at 6 months of corrected gestational age than did control subjects [7]. Heart rate, respiratory rate, oxygenation, blood glucose, sleep patterns, and behavior observed in preterm/LBW infants held skin-to-skin are either similar to or better than in infants separated from their mothers [16–18]. The level of stress, indicated by salivary cortisol, was found to be lower in newborn infants held in SSC [19]. Cognitive development seemed to improve with KMC [20]. Various studies have shown that KMC may reduce maternal anxiety and improve self-efficacy and mother-child bonding, which may result in more effective newborn care [21–26].

The evidence summarized above comes from studies where KMC was initiated in the hospital [27]. There has been considerable interest in KMC initiated outside health facilities for LBW babies born at home or discharged early from health facilities. There is only one published randomized controlled trial of community-initiated Kangaroo Mother Care (cKMC) [28]. In that cluster randomized controlled trial conducted in Bangladesh, cKMC was promoted for all newborns, regardless of birth weight. In intervention clusters, about 77% of infants received any SSC, with only 24% receiving any SSC for >7 h/day in the first 2 days of life. The study reported no overall difference in mortality between the intervention and control groups (OR 1.06, 95% CI 0.76–1.48). Although there was a reduction in mortality among babies with birth weight ≤2000 g (OR 0.37, 95% CI 0.16–0.86), mortality was higher (OR 1.31, 95% CI 0.94–1.82) among infants with missing birth weights (about 40% of all newborns, and 65% of all deaths had a missing birth weight). The authors concluded that there was insufficient evidence to support implementing cKMC.

In most developing countries, only a small proportion of LBW infants can be hospitalized for care. In these settings, a large proportion of deliveries still take place at home, and even if born in facilities, newborns are discharged early. It is therefore crucial to evaluate cKMC in a well-conducted efficacy study.

We are undertaking a randomized controlled trial to evaluate the impact of cKMC on newborn survival. In preparation for this study, we undertook intensive formative research to develop the cKMC intervention package to assure ourselves that there would be a high rate of cKMC adoption by families.

This paper describes an efficacy trial in which the primary objective is to determine the impact of promoting cKMC for LBW infants on postenrollment neonatal mortality and on mortality from enrollment up to 6 months of age. The secondary objectives of the study are to determine the impact of promoting cKMC on the following:

- Proportion of infants exclusively breastfed at 1 and 3 months of age
- Weight, length, and head circumference gain at 1, 3, and 6 months of age
- Incidence of infections and hospitalizations in the neonatal period and between 1 month and 5 months of age
- Recognition of illness and early care seeking by the family from appropriate sources
- In a subsample, neurodevelopment at 6 and 12 months of age

## Methods/design

The protocol has been prepared according to the Standard Protocol Items: Recommendations for Interventional Trials (Additional file 1).

### Study design

The study is a randomized controlled trial. The first 550 infants enrolled are also enrolled in a study whose aim is to assess the impact of the intervention on neurodevelopment (ClinicalTrials.gov identifier NCT02631343). In the formative research conducted prior to trial initiation, qualitative research methods such as in-depth interviews, focus group discussions, and observations were used to ascertain practices around birth and to assess the feasibility and acceptability of cKMC. A prototype intervention package and delivery strategy were designed, and household trials were conducted to ascertain adoption rates among mothers of LBW babies [29].

### Study setting

The study is ongoing in Palwal and Faridabad districts in the State of Haryana, India. In this region with a population of around 2 million, the median family size is 6 (IQR 4–9) [3]. The birth rate is 25.6/1000 [30]. Forty percent of deliveries occur at home, and 24% of all babies are born with LBW (<2500 g) [3]. The common occupations for men are employment in nearby factories or other commercial enterprises (38%), daily wage laborers in construction (20%), self-employment (22%), farming (10%), and government service (5%); 5% are unemployed. The median number of years of schooling is 8 for men (IQR 5–11) and 5 for women (IQR 0–8). Over 40% of women have never been to school, and almost all (95%) women do not work outside the home for an income [3].

The common sources of drinking water are hand pumps (approximately 40%), piped water supply (approximately 30%), public taps (approximately 20%), and others (10%). Around 60% of the population uses open fields for defecation. A high proportion (92%) of families has electricity in their homes. Care of ill children is most often (60%) sought from private practitioners within and outside the villages.

The government-owned primary health center (PHC) is the most basic structural and functional unit of the health system. Apart from regular medical treatment, PHCs focus on maternal-child health promotion, including family planning as well as safe water supply access and basic sanitation. A PHC caters to a population of approximately 30,000. Under each PHC, there are 4 to 5 subcenters, each of which caters to a population of approximately 5000. Each subcenter is managed by a female health worker commonly known as the *auxiliary nurse midwife* (ANM) [31]. Her role encompasses promotion of preventive and curative health services, which include family-planning services, nutrition education, health education, collaborative services for improvement of sanitation, immunization for control of communicable diseases, treatment of minor ailments and first aid in emergencies, organizing health days in the community, and working closely with Accredited Social Health Activists (ASHAs) [31]. Each ANM is supported by four or five ASHAs, who are the first contact persons between the community and the health system (http://nrhm.gov.in).

ASHAs are community health workers employed by the government and cater to a population of approximately 1000. They are local women trained to act as health educators and promote universal immunization, timely referral, and escorting women and children for reproductive and child health and other health care programs in their communities [32].

Besides these workers, Anganwadi workers (AWWs) operate from Anganwadi centers (AWCs). An AWC is a government-sponsored child care and maternal care center in India. It caters to children in the 0–6 years age group. This center is the focal point for delivery of all services under the Integrated Child Development Services program to children and women (http://icds-wcd.nic.in/icds/icds.aspx). One AWW caters to a population of approximately 1000.

### Study participants

We are enrolling babies between 1500 and 2250 g because our formative research revealed that most babies weighing more than 2250 g did not want to remain in the KMC position and tried to wriggle out as early as 4–5 days after initiating KMC. Babies weighing <1500 g are often unstable and have feeding and breathing difficulties, and including them may raise safety concerns, especially in a study that is being conducted in the community. Because in this study KMC is initiated at home, and because two-thirds of deliveries take place in hospitals where the recommended practice is to discharge mothers after 48 h, the window for enrolling babies is within 3 days of birth.

### Inclusion and exclusion criteria

All LBW babies (1500–2250 g) and their mothers are screened within 3 days of delivery. Babies born at home and babies born in health facilities are included if KMC was not initiated in the facility. Infants who are unable to feed, have breathing problems, have gross congenital malformations, or are less than normally active (i.e., babies whose movements are less than usual or who do not wake up with stimulation or during assessment on the day of the visit) are referred to hospitals. Those mothers intending to move away over the next 6 months or who do not consent to participate are also excluded.

### Sample size

Approximately 12% of infants in the study area weigh between 1500 g and 2250 g [3]. Assuming a postenrollment neonatal mortality risk of 42 per 1000 in this birth weight group, and accounting for 10% attrition, we need to enroll a total of 10,500 infants (with 95% confidence and 90% power) to detect a 30% relative mortality reduction among neonates. We expect that this will also enable us to detect impact on mortality from enrollment until 6 months of age. The data safety and monitoring committee (DSMC) will review the sample size when about half of the enrollments have been completed, with the possibility of adjusting the sample size.

### Identification of pregnancies by the surveillance team

Surveillance workers conduct door-to-door surveys in the study areas. Surveys are redone at least quarterly. The details of all pregnant women are communicated to a study coordinator who allocates them to the relevant worker for follow-up.

### Pregnancy follow-up, screening, and enrollment

All identified pregnancies are allocated to the pregnancy follow-up and screening and enrollment (PSE) team. Each PSE team member covers a fixed geographical area comprising 3500–5000 households. These workers keep in touch with pregnant women through phone contacts or home visits (if phone calls are unsuccessful) monthly in the initial months, twice weekly in the third trimester, and daily as the time of delivery approaches. The study is explained to families, and they are asked to inform the study team when the woman delivers. Outcomes of pregnancies—abortions, stillbirths, and live births—are documented. If a woman delivers at home, the team visits the household as soon as possible after delivery and weighs the infant. Workers keep in touch with families of women who deliver in the hospital, and women are visited as soon as possible after discharge. Prior to screening, consent for participation is taken. If the weight of the baby is <1800 g, families are counseled to visit health facilities as mandated by the Indian government [33].

Infants who are identified as being ill are referred, and contact with the family is maintained. Hospitalization is facilitated by putting the family in touch with the ASHA in their area. The baby is screened for enrollment when discharged from the hospital if still within the 3-day enrollment window.

### Randomization, allocation, and masking

The randomization list was prepared using random permuted blocks of variable block size by an offsite statistician from the WHO in Geneva who is not otherwise involved with the trial. Once an infant meets the inclusion criteria, has no exclusion criteria, and the family consents to participation, the PSE worker calls the coordinator in charge for allocating the participant an identification number. The coordinator opens a sequentially numbered, opaque sealed envelope (SNOSE) next in serial order and communicates the participant identification number to the worker. These procedures are identical for intervention and control participants, and PSE workers are not aware of the group allocation.

For twins, triplets, or multiple children (i.e., children of parents living in the same household enrolled earlier and randomized to the intervention group), the same intervention is allocated, and the participant identification number is suffixed with letters B and C, respectively. Similarly, if the mother has another eligible baby from a subsequent pregnancy, the same identification number is allocated, and a suffix is added. Only if the previously enrolled infant is in the control group is a new SNOSE opened. This strategy is being followed to give the second infant a chance of getting the intervention.

### Home visits by ASHAs under the national program

Under the national program, all infants (in the intervention or control group) are to be visited on days 1, 3, 7, 14, 21, 28, and 42 for counseling on essential newborn care, identification of illnesses, and referral of ill infants [34]. The national program also envisages that additional visits will be required for babies born with LBW. The visits by the team that supports the mother to do KMC have also been scheduled with what is visualized in the national program kept in mind (days 1, 2, 3, 5, 7, 10, 14, 21, and 28).

### Intervention delivery

The coordinator informs the intervention delivery team whenever an infant is allocated to the intervention group. This team comprises workers with educational and work experience backgrounds similar to those of government ANMs and ASHAs, and therefore they are designated as study ANMs and study ASHAs. A pair of workers makes the first visit to explain KMC and support the mother to do it. KMC is promoted using the local term identified during formative research—*chaati se chipkana*—or “sticking the baby to the chest.” The team uses photographs, helps the mother place the baby in the KMC position, and observes breastfeeding. The mother is advised to do SSC in the position in which she is most comfortable: semireclining or supine. Family members, including husband and mother-in-law or other relatives in the home, are also taught the procedure and encouraged to do SSC during the periods when the mother is not doing so or by helping with the household work so that the mother is free to be with the baby.

As described above, the visits are made daily for the first 3 days, on days 5 and 7, twice in week 2, and once each in weeks 3 and 4. ANMs and ASHAs visit together on the first 3 days. Subsequently, the ANM visits if the mother is having problems in doing KMC or needs counseling for breastfeeding. Visits end at 28 days or earlier if the baby no longer wants to be kept in KMC position as evidenced by the baby trying to wriggle out. At each visit, the ASHA documents how much KMC has been given since the last visit.

### Outcome ascertainment

Enrolled infants in both the intervention and control groups are visited by an independent outcome ascertainment team. An attempt is made to keep the team blinded as far as possible to whether the baby belongs to the intervention or control group. Weight, length, and head circumference are measured by a pair of workers. A baseline form containing socioeconomic and demographic characteristics is completed. Outcomes are also ascertained at 1, 3, and 6 months of age. At each of these visits, child care practices, prevalence of illness, and details of treatment-seeking and hospitalizations are ascertained. Additionally, at the month 1 visit, information is sought on whether any SSC was given and, if so, the number of days and the number of hours each day SSC was given by the mother or any other family member. Weights and length measurements are taken using a digital hanging weighing scale (AWS-SR-20; American Weigh Scales, Cumming, GA, USA) and an infantometer (model 417; Seca, Chino, CA, USA), respectively; head circumference is measured with head-measuring tape (model 212; Seca). At their scheduled visits, the outcome ascertainment team also documents visits made by government workers for all enrolled infants.

### Verbal autopsies

A member of a separate team well trained in verbal autopsy techniques visits each household as soon as possible after learning of the death of a study infant. A verbal autopsy questionnaire is completed; this technique is used to ascertain the cause of death [35]. An interview is carried out with family members of the deceased infant by using a structured questionnaire to elicit signs and symptoms and other pertinent information, which are used to assign the probable cause of death [35].

### Process evaluation

Activities conducted by different teams are observed by the process evaluation team. These include counseling by the intervention delivery team, pregnancy surveillance, pregnancy follow-up, screening and enrollment, and anthropometric measurements. All main activities and individual team members are covered. Feedback is given to the person concerned, and corrective action is taken if necessary.

### Training and standardization of study teams

All staff were trained in study objectives, overall strategy, and good clinical practice [36]. Each team underwent intensive training pertaining to their work areas (e.g., pregnancy surveillance, door-to-door surveying, complete documentation of household members, reporting pregnancies, and taking birth weights). The intervention delivery team was trained in counseling mothers in SSC and in lactation support. They also visited hospitals with KMC wards for hands-on practice sessions.

For the outcome measurement team, weight, length, and head circumference inter- and intraobserver standardization exercises were conducted before study initiation and are repeated every 6 months. Weighing scales and infantometers are calibrated periodically using standard weights and length measurement rods.

### Data management and monitoring

All data are captured electronically on phones or tablets. Range and consistency checks are built in. Queries are returned to the respective teams and resolved within 1 week of data collection. Data are transferred monthly to the WHO for offsite data quality checks.

### General principles for analysis

The primary analyses will be conducted on an intention-to-treat basis. Analyses will be performed with both person time (infant years of follow-up, primary analysis) and number of babies as the denominator. Random effects models and robust standard errors will be used to account for clustering of deaths for twins, triplets, and other study babies living in the same household. Data cleaning will be completed and the databases locked before the data analysis workshop. The code will be broken after the analysis has been carried out and we interpret the findings, aiming to reduce the risk of prejudice.

### Flow of participants

**Fig. 1:**
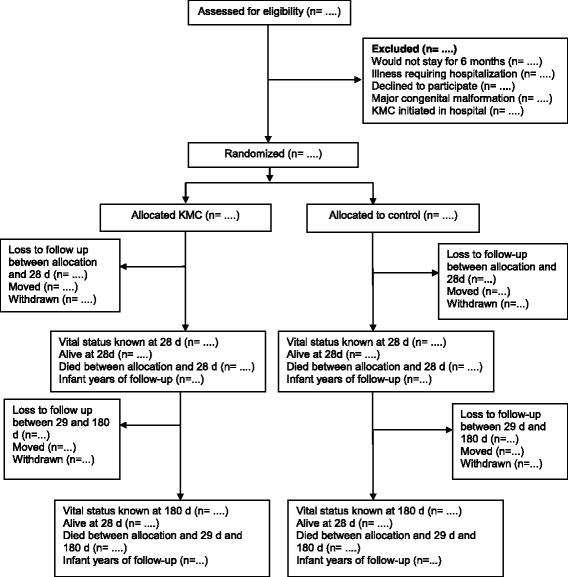
Participant flow through the study. *KMC* Kangaroo Mother Care

The flow and number of participants through assessment of eligibility, randomization, follow-up, and analysis will be documented (Fig. 1). Reasons for exclusions and withdrawals will be described.

### Comparability of participants in the two groups

Summary values (means, proportions) for background characteristics in the intervention and control groups will be presented in a baseline table. These data will represent maternal age, parity, maternal education, wealth index of the household, place of delivery, singleton or multiple birth, sex of infant, birth weight, and age at enrollment. As per the Consolidated Standards of Reporting Trials (CONSORT) guidelines, we will not perform significance tests to compare baseline characteristics between the study arms [37, 38]. Although our large sample size is likely to yield balance between the two study arms in the main analyses, in our planned subgroup analyses, we will carefully evaluate the size of any baseline imbalance. Imbalanced characteristics that predict death will be appropriately adjusted for statistically.

### Main effects

For the primary outcome (all-cause mortality between enrollment and 28 days of age), data will first be analyzed using person time as the denominator. Hazard Ratios ﻿(HRs) and 95% CIs will be calculated using a Cox regression model to compare the effect of the intervention (cKMC) with the control condition on infant deaths. We will also estimate the effect of cKMC using the number of enrolled infants as the denominator to deduce risk ratios (RRs) using generalized linear models (GLMs) of the binomial family with a log-link function. However, if these regression models do not converge, the delta method for nonlinear combinations of estimated parameters from the coefficients computed in logistic regression shall be used [39]. The percentage efficacy of the intervention will be calculated as (1 − HR) × 100 and (1 − RR) × 100. To estimate the number needed to treat, the reciprocal of the risk difference, we will estimate the latter with a GLM of the binomial family with an identity link. In the unlikely event that there is imbalance between the intervention and control groups, the final statistical models will include appropriate statistical adjustment for all imbalanced variables. The effect of cKMC on all-cause mortality between enrollment and 180 days of age will be assessed using the approaches outlined above.

The effect of KMC on secondary outcomes (exclusive breastfeeding, weight, length gain, incidence of important illnesses and hospitalizations, and care-seeking) will be assessed using linear or logistic regression, as appropriate, adjusting for clustering in case of twins or another enrolled baby subsequently born to the same woman, as well as other potential confounders, and taking clustering into account as in the primary analysis. Though an extended cost-effectiveness analysis is planned, it is beyond the scope of this paper and will be described in a separate paper.

### Subgroup analysis

We will undertake subgroup analyses for infants according to birth weight categories, sex, timing of initiation of KMC, and duration of KMC. The relative measures of effect within each of these subgroups will be estimated.

### Data and safety monitoring committee

A DSMC has been constituted to review the data twice a year with at least one face-to-face meeting every year to monitor the progress of the trial and assess the safety of the intervention. The members include an epidemiologist, a statistician a neonatologist and an expert on community interventions. The DSMC examines deaths, rate of enrollment and compliance to the intervention every 6 months. An interim analysis will be conducted when approximately 50% of the infants have been enrolled. This analysis will be conducted in a blinded manner (treatment groups X and Y). The randomization code shall be broken following the recommendation of the DSMC if there is clear evidence of a difference in mortality between the two groups. The committee will advise the study team on study continuation, modification or termination based on preestablished stopping rules.

### Study timeline

**Fig. 2:**
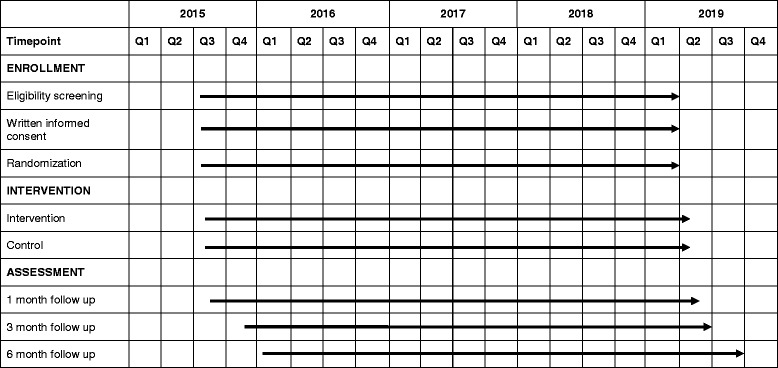
Standard Protocol Items: Recommendations for Interventional Trials (SPIRIT) figure

The study is running from July 2015 to September 2019 (Fig. 2).

### Monitoring visits

Technical staff from the WHO interact with the study team through conference calls and visits to the site to review study progress. Monthly reports are shared by the study team. All key areas are monitored; these include the enrollment rate, timing of start of the intervention delivery, consent procedures, follow-up visits, and timely transmission of data to the WHO.

## Discussion

This study will provide information on the impact of KMC initiated at the community level. So far, the experiences and documented benefits of KMC come from hospital settings where KMC was initiated with the assistance of skilled health workers. In a Cochrane review, the studies were done in hospital settings, except for one study conducted in the community in Bangladesh [28]. That community-initiated study found limited adoption of KMC by mothers. Besides, birth weights were known for only 60% of the babies. There was no measurable impact of KMC on mortality. Therefore, the question whether benefits of KMC seen in hospital settings can be translated to settings where KMC is initiated in the community in low- and middle-income countries remains unanswered.

Formative research showed that cKMC was feasible and acceptable, and high adoption rates were observed in mothers of LBW babies. However, skilled counseling was critical to resolve barriers and achieve these rates. We feel that the initiation of KMC requires a worker from a higher cadre (e.g., an ANM) than community-level health workers such as ASHAs. The follow-up support could be provided by ASHAs. In this trial, therefore, local workers equivalent to government ANMs were selected for initiation of the intervention. Well-trained community health workers equivalent to the government ASHAs provide follow-up support, with the ANM visiting for problems. Because this is the first time cKMC is being implemented in a community setting, a design using well-trained study workers is considered to be most appropriate, owing to there being a need to estimate the efficacy of the intervention.

On the basis of standard KMC guidelines, mothers and other caregivers in this trial are advised to continue KMC until 28 days of age or until the baby no longer wants to stay in the KMC position [6, 33]. The primary outcome is postenrollment neonatal mortality and postenrollment mortality until 6 months of age. Attempts are made to initiate the intervention as soon as possible after birth because a large proportion of the neonatal mortality occurs within the first 24 h.

We believe that contamination between the mother-infant pairs randomized to cKMC promotion and those in the control families is unlikely. The households with babies meeting the inclusion criteria are far away from each other. Further, babies born in households where a baby has previously been treated with KMC is not randomized and will be provided KMC. Besides, we did not encounter any resistance to the practice from community members or health workers during formative research, which would have necessitated the need for community awareness generation activities. Because promotion of cKMC requires hands-on demonstration, intensive counseling, and high-level motivation, it is unlikely that mothers or other family members will practice KMC without adequate support.

Although it may be deemed that we are implementing cKMC with high intensity in our trial, we believe that the intervention is scalable in similar settings because the workers delivering the intervention are similar in terms of education and experience to their government-employed counterparts. Moreover, there will be relatively few babies who need KMC, so it should be possible to manage them with the existing number of government services. The process evaluation activities will provide insights into barriers and facilitating factors. These will be valuable sources of information for later scale-up and adaptation to other settings.

## Trial status

The trial is ongoing. Participant recruitment is expected to be completed by mid-2019.

## Additional file

Additional file 1: Standard Protocol Items: Recommendations for Interventional Trials (SPIRIT) checklist. (DOC 122 kb)

## Abbreviations

ANM: Auxiliary nurse midwife
ASHA: Accredited Social Health Activist
AWC: Anganwadi center
AWW: Anganwadi worker
cKMC: Community-initiated Kangaroo Mother Care
CONSORT: Consolidated Standards of Reporting Trials
DSMC: Data safety and monitoring committee
GLM: Generalized linear model
KMC: Kangaroo Mother Care
LBW: Low birth weight
PHC: Primary health center
PSE: Pregnancy follow-up and screening and enrollment
RR: Risk ratio
SNOSE: Sequentially numbered, opaque sealed envelope
SPIRIT: Standard Protocol Items: Recommendations for Interventional Trials
SSC: Skin-to-skin contact
WHO: World Health Organization
